# Disability, Quality of Life, and Vitalism in End-of-Life Care

**DOI:** 10.1007/s10730-025-09561-w

**Published:** 2025-10-15

**Authors:** Devan Stahl

**Affiliations:** Baylor University, One Bear Place #97284, Waco, TX 76798-7284 USA

**Keywords:** Disability, Vitalism, Quality of life

## Abstract

This article draws upon the work of disability bioethicists to offer additional reflections on how and why conflicts sometimes emerge between the surrogates of disabled patients and health care practitioners. The Best Interest Values (BIV) system described by Fiester can lead to, or coincide with, ableist attitudes that endanger the lives and dignity of disabled people. At the same time, her competing hierarchies could do a better job distinguishing quality of life concerns from vitalism, the latter of which does not well represent many disability advocates’ concerns regarding healthcare systems. The article concludes by arguing that acquiescing to surrogate demands and legal requirements to prolong the lives of disabled patients can lead to inhumane treatment plans that ought to be resisted by health care practitioners.

Disability advocates have long worried that health care practitioners (HCPs) undervalue their lives and underdetermine their quality of life (QoL) (Koch, 2004; Mukherjee et al., 2022; National Council on Disability, 2019). As a result, many patients with disabilities and their surrogates may resist efforts to limit, withhold, or withdraw life sustaining treatments for fear that physicians’ bias against disability may prematurely end their lives. This resistance can then lead to conflicts over end-of-life treatment plans. In her essay, “Surrogate Wars: The ‘Best Interest Values’ Hierarchy & End-of-Life Conflicts with Surrogate Decision-Makers” (2025), Autumn Fiester details such conflicts, which she argues have persisted for decades, between HCPs and Surrogate Decision Makers (SDMs) in end-of-life care. Fiester believes these debates have become nearly intractable because HCPs and a sizeable minority of SDM hold different sets of values surrounding what makes for a good life. Fiester argues that healthcare institutions ought to more constructively engage stakeholders who value continued interventions despite a patient’s poor prognosis even in cases in which continued care is judged to be “inappropriate,” “nonbeneficial,” or “futile” (2025, p. 440). Ultimately, Fiester suggests that HCPs should accommodate surrogate demands more frequently than they currently do to avoid values imposition on SDMs, who are in a less powerful position than HCPs within the health care system.

Fiester’s suggestion that HCPs defer to surrogates more often when they request life sustaining treatments will be well-received by many families. As Fiester points out, a sizeable minority of surrogates hold “Life-Continuation Values” the first two tenets of which are the belief that “1. Each human life should be extended as along as possible, with maximizing the quantity of life being the ultimate goal of medical interventions” and “2. Human life has intrinsic worth in any state or condition, so being alive is always a good and being ‘alive’ means having a heartbeat" (2025, pp. 449–450). Such beliefs have sometimes been dubbed “vitalism” in bioethics literature (Keown, 1998, p. 256). Vitalism conflicts with what Fiester dubs HCPs’ “Best Interest Values” which hold that some conditions, including disabilities, render life worse than death (pp. 450–451). If certain disabilities are thought to drastically lower a person’s quality of life, then HCPs may be less willing to pursue aggressive treatments for persons with disabilities. Research shows, however, that while many people, including physicians, imagine that disability make lives poor or even unlivable, people with disabilities, even seriously life-limiting disabilities, rate their QoL similarly as those without disabilities (Albrecht & Devlinger, 1999; Gerhart et al., 1994; Kuzma-Kozakiewicz et al., 2019; Rousseau et al., 2015; Skotko et al., 2011). Many people with disabilities and their surrogates will request aggressive medical treatments to continue lives they believe are worth living.

At the same time, this does not mean that people with disabilities would desire to live no matter the state of their health or functionality. It would be a mistake to conflate the concerns of all disability advocates with a preference toward vitalism, or the valuing of life in any state. Vitalism does not well represent many disability advocates’ concerns regarding ableism within health care. This essay argues that both the BIV and LCV hierarchies that Fiester describes can do serious harm to disabled people when certain treatments are denied to them or forced upon them. For this reason, acquiescing to surrogate demands to prolong the lives of disabled patients can sometimes lead to treatment plans that ought to be resisted by HCPs.

## Quality of Life Judgments and Disability Bias

Although there is no consensus on how to measure or universally define “quality of life” within medicine, QoL considerations are understood by many to be an important consideration when deciding which interventions or services are most likely help a patient live well and in accordance with their preferences and values (Stahl, 2022). A treatment can be successful in terms of physiologic effect while at the same time rendering an individual unable to participate in activities that give his or her life meaning. Shared decision making between HCPs and patients/SDMs is predicated on the idea that treatment plans ought to be tailored to patient values and preferences (Dy & Purnell, 2012). Yet, it would be a mistake to assume that all HCPs are well-equipped to imagine or even collaborate with patients and SDMs regarding which domains are most important for the patient’s QoL or well-being (Erez & Gal, 2020; Janse et al., 2004). Consequently, when QoL is judged by someone other than the patient or without consideration to what gave the patient pleasure or purpose, implicit biases against disability can lead to conflicts between patients/surrogates and HCPs.

Quality of life judgments in medicine have garnered much attention by disability advocates. The gap between how disabled people and HCPs judge their QoL has been dubbed the “disability paradox” and its effects have been researched for decades (Albrecht & Devlieger, 1999; Drum et al., 2008; O’Hara et al., 2021; Ubel et al., 2005). Such research consistently shows that people with disabilities, even disabilities that physicians believe are severe, rate their QoL similarly to be people without disabilities. Despite these findings, a recent study revealed that 82% of physicians believe people with significant disabilities have a worse QoL than people without disabilities (Iezzoni et al., 2021). Both disability advocates and the National Institutes for Health worry that the disability paradox can result in implicit biases that contribute to health disparities for people with disabilities (NIMHD, 2023).

Health researchers suggest that disability bias can have at least four negative effects on patient care. According to Haque and Stein (2020, pp. 287–288), disability bias can result first in increased paternalism when HCPs assume patients with disabilities are less competent than non-disabled patients. Second, HCPs often inaccurately assume their disabled patients are suffering, leading to more conservative treatment plans because they do not want to give patients more than they “can handle” (Haque & Stein, 2020, p. 287). Third, patients with disabilities are often believed to be friendlier or more prosocial, leading to a presumption they would be less inclined to want scarce resources or would be more likely to give up their lives to save others when medical treatments are being rationed (Haque & Stein, 2020, p. 287). Finally, HCPs can have a lower threshold for withholding and withdrawing care for their disabled patients, because they judge a pre-existing disability to have a more catastrophic effect on a patient’s life than the patient experiences (Haque & Stein, 2020, p. 288).

Of particular concern for many disability advocates is that disability biases, such as those named above, can result in meaningful lives being cut short. Arguments over what constitutes medical “futility” have been a primary target for disability advocates in debates over end-of-life care, particularly when HCPs use futility judgments to override patient or surrogate requests for continued life sustaining treatments. There is evidence that physicians who believe that their patients have a low QoL or are “suffering” from disability are more likely to judge aggressive care “futile” and recommend comfort care (Cruz-Oliver et al., 2010; Haque & Stein, 2020; National Council on Disability, 2019; National Disability Rights Network, 2022). In its Report “Medical Futility and Disability Bias” the National Council on Disability (NCD) writes:

It has been well-documented that healthcare providers significantly undervalue life with a disability, in part because most medical education does not include accurate information on the lived experiences of people with disabilities. As a result, healthcare providers remain largely unaware of the high quality of life and happiness that many people with disabilities experience. This lack of awareness has impacted medical futility decision making and, in some cases, robbed people with disabilities of their chance to recover (2019, pp. 9-10).

The NCD Report cites futility debates from the 1990s and early 2000s in which some bioethicists argued for a category of “qualitative futility” that would allow physicians to withhold or withdraw a treatment they believed would not result in an acceptable QoL for the patient (Schneidermann et al., 1990, pp. 952–953). Schneidermann, Jecker and Johnson, who first proposed the category of qualitative futility, were careful to note that treatments should not be withdrawn from disabled patients who could continue to achieve their life goals and that in such cases, “[disabled patients] (or their surrogates) have the right to receive or reject any medical treatment according to their own perceptions of benefits compared with burdens” (1990, p. 953). The NCD however, argues qualitative futility judgments too easily allow for biased perceptions regarding what kinds of lives of worth living (2019, p. 23).

The NCD goes on to argue that even judgments regarding “quantitative futility” and can be influenced by disability bias (2019, pp. 23–24). Quantitative futility is typically understood as treatments with a very low probability of succeeding, with Schneidermann, Jecker, and Johnson agreeing it would qualify if a treatment had less than a 1% chance of succeeding (1990, p. 952). Researchers have shown, however, that many physicians who unilaterally write Do Not Attempt Resuscitation orders based upon quantitative futility judgments estimate the patient’s chances of survival are well above the agreed upon 1% threshold proposed by Schneidermann, Jecker, and Johnson (Curtis et al., 1995). While most disability bioethicists do not believe HCPs should be required to provide treatments that will not have the desired physiological effect (Francis & Silvers, 2007; Shepherd, 2006; Stahl, 2022) implicit biases against disability can impact how physicians judge the probable success of a treatment (Cruz-Oliver et al., 2010).

In response to QoL concerns by disability advocates, states such as Texas have amended their Health and Safety Code (166.046) to ensure that physicians and ethics committees are barred from using their own subjective QoL judgments as a reason to override a patient or surrogate’s request to continue life sustaining treatments. Hospitals’ decision to withdraw life sustaining treatment must be made “without regard to any judgment on a patient’s quality of life” (HB 3162). Bioethicists and HCPs who seek to reduce disability bias in health care may find Texas’ statutory amendment an important step in reducing conflicts between HCPs and SDMs in end-of-life care for disabled patients. Fiester, for example, names QoL judgments as a significant area in which conflicts are likely to emerge between HCPs and SDMs, and in large part because they tend to judge disability differently.

Fiester argues that most HCPs adhere to a “Best Interest Values” (BIV) hierarchy wherein “medically inappropriate” treatment can and should be withheld from patients. Fiester names eight normative commitments as constitutive of BIV, two of which are value judgments against disability. First, the BIV system holds that, “meaningful existence is incompatible with severe cognitive impairment or significant intellectual disability” (p. 446) and second, “meaningful existence is incompatible with severe physical impairment or disability” (p. 446). HCPs who adhere to the BIV system, which Fiester describes as “ubiquitous” in health care, place a high value on what they understand as QoL and take a rather rigid stance on what constitutes a high QoL. Moreover, they are more likely to believe that physical dependence, which often accompanies physical disability, is “deplorable” (p. 446).

In contrast, Fiester believes that a sizable minority of patients and surrogate decision makers adhere to what she calls “Life Continuation Values” (LCV). Although none of the values in this hierarchy names disability directly, the LCV system rejects QoL judgments made by HCPs in end-of-life care: “quality of life judgments should not be made to determine who lives or dies” (p. 450). According to Fiester, those who hold LCV do not believe that these QoL judgments are within the scope of clinical expertise; rather, they are value judgements about lives that are worth living. Many disability advocates similarly argue that QoL is a subjective judgment that should only be made by patients and, if necessary, their surrogates (National Council on Disability, 2019).

Perceived quality of life conflicts in end-of-life care like those Fiester describes took center stage in the case of Michael Hickson, whose story made national headlines in 2020. Hickson, who had previously suffered a cardiac arrest that resulted in disabilities, including an anoxic brain injury, blindness, and quadriplegia, died after his clinical care team decided not to place him on a ventilator after he contracted Covid-19. In an exchange that Hickson’s wife, Melissa Hickson, audio-recorded, Hickson’s physician explained that aggressive treatment would be “futile” because Michael didn’t have much “quality of life.” Seeking clarification, Mrs. Hickson asked if the physician was referring to her husband’s pre-existing paralysis and brain injury, to which his physician replied “correct” (Shapiro, 2020).

Of course, the hospital treating Hickson was unable to comment directly upon his case to the media and it is unclear whether Michael Hickson could have survived his illness if placed on a ventilator. Yet, even if the audio recordings between Mrs. Hickson and Mr. Hickson’s treating physician were taken out of context or somehow fail to capture a more nuanced view held by Mr. Hickson’s treating physician, Mrs. Hickson clearly believed that she and her husband’s treatment team had different understandings of Mr. Hickson’s quality of life. Mrs. Hickson believed her husband had a good quality of life, that if he could speak for himself, he would have wanted treatment, and that he was discriminated against because of his disability (Shapiro, 2020). Mrs. Hickson could have been fundamentally mistaken about who her husband was and what he valued, but there is little reason to believe that Mr. Hickson’s medical team, who had never met him prior to his hospitalization, would have had a better understanding of who Mr. Hickson was and what he valued than his wife. According to Fiester, in cases such as these, HCPs should yield considerable power to families to articulate the disabled patient’s QoL and what sorts of end-of-life care the patient would want.^1^

## Disentangling Quality of Life and Vitalism

It is important to distinguish disability advocate’s concerns over HCPs’ quality of life judgments with vitalism, or a preference for life in any state. Certainly, there are people, including disabled people, who are vitalists and would request continued life prolonging treatment to extend their life at any cost. And while there is certainly overlap between a rejection of QoL judgments in medicine and vitalism, it is logically coherent to believe that HCPs place the threshold for an acceptable QoL too high for many disabled people while also believing that some states of life are in fact worse than death.

Fiester references the legal drama over Terri Schiavo, a young woman who was diagnosed as being in a permanent vegetative state (PVS) after she suffered a cardiac arrest and anoxic brain injury, as a case in which the BIV and LCV hierarchies were in clear conflict. Fiester argues that those who hold Life Continuation Values are likely to agree that “human life has intrinsic worth in any state or condition, so being alive is always a good…” and “each human life should be extended as long as possible, with maximizing the quantity of life being the ultimate goal of medical interventions” (2025, p. 450). Ms. Schiavo’s parents, the Schindlers, seemed to hold such values as they argued that her artificial hydration and nutrition should be maintained so that she could live. In contrast, HCPs are more likely to agree that “nonexistence can be preferable to existence, i.e., being dead can be better for a person than being alive” (Fiester, 2025, p. 445). Her husband Michael Schiavo argued that Terri Schiavo held this view and that she would not have wanted to continue living in a persistent vegetative state (PVS).

Disability bioethicists have long warned the dangers of conflating disability discrimination with support for vitalism. Using the Terri Schiavo case as an example, disability scholars Leslie Pickering Francis and Anita Silvers argue that various versions of vitalism fail to protect people with intellectual disabilities from harm and are likely to lead to problematic paternalism for disabled patients (Francis & Silvers, 2007). A “straight vitalist” perspective, which argues for the continuation of all life sustaining treatment, may lead to the continuation of treatments even when a formerly competent adult issued prior preferences against such treatment (Francis & Silvers, 2007, p. 817). This, they argue is “moralist paternalism,” which imposes values upon disabled patients they never held prior to their hospitalization.

Francis and Silvers agree that HCPs should presume, with some important exceptions detailed below, that patients would want to live in the absence of evidence they would not. They also argue, however, that when deciding upon treatments for incompetent patients, HCPs and SDMs should employ a “broad voluntarist exception” that would allow for more evidence than the direct testimony of the patient to override the default position of preserving life. For example, in the case of Terri Schiavo, the court scrutinized evidence that she would not have wanted to continue to live in PVS when her surrogate, Michael Schiavo, requested withdrawal of her artificial hydration and nutrition. Although Ms. Schiavo’s parents, the Schindlers, claimed that she was a Roman Catholic who agreed with the Church’s stance on continuing life sustaining treatment in PVS, they were unable to present evidence that Ms. Schiavo remained a committed Catholic as an adult, or supported the Catholic position on life sustaining treatment (Pearse, 1998). On the contrary, the court determined that there was “clear and convincing” evidence based on the testimony of several credible witnesses that Ms. Schiavo would not have wanted to live in PVS (Pearse, 1998, p. 13). Francis and Silvers (2007) conclude that in Ms. Schiavo’s case, allowing her parents’ religious values or preference for vitalism to supersede her clear prior values and preferences would have been paternalistic and inappropriate.

Vitalism, which sees life itself as a paramount value, can impose values upon people with disabilities they may never have held or ignore who they are entirely. The danger of confusing critiques of QoL with vitalism is that individual disabled patients’ lives and experiences can be ignored when sweeping generalizations are made about their values or preferences for medical care. It is entirely possible that a disabled patient could believe their HCP’s recommendation to limit life sustaining treatment comes from an implicit bias against their lives generally, while still preferring not to receive maximally aggressive medical treatment, even if it were life-prolonging.

Disability advocates’ involvement in the case of Terri Schiavo complicates the distinction between disability concerns over QoL and vitalism, however. During the decades-long legal battles over Terri Schiavo’s medical treatment, many disability advocates sided with the Schindlers, who wanted to keep Ms. Schiavo alive through artificial hydration and nutrition. In 2003, twelve disability rights groups filed an amicus brief (Brief of Amici Curiae Not Dead Yet et al., 2004) with the Florida Supreme Court to remove Michael Schiavo as Ms. Schiavo’s surrogate. The groups argued that Ms. Schiavo was being discriminated against because she was disabled. If her husband was allowed to remove her life sustaining medical treatment, this would open the door for future third parties to limit life sustaining treatments to disabled people when they judged the individual’s quality of life to be too low (Brief of Amici Curiae Not Dead Yet et al., 2004, p. 1). The Schindler’s similarly argued that Ms. Schiavo was disabled and that her husband and HCPs wanted her to die because she was disabled (Hannigan, 2006).

Terri Schiavo did meet the definition of a person with a “disability” under the Americans with Disabilities Act (1990) since she had a physical or mental condition that substantially limited a major life activity. Yet, it does not necessarily follow that the decision to remove her artificial hydration and nutrition constituted disability discrimination. Michael Schiavo did not argue that his decision to withdraw treatment was based upon his personal judgments about his wife’s quality of life. Instead, he argued that Ms. Schiavo would not have wanted to live in PVS. In other words, it was her prior judgment that a life in PVS was not worth living. Ultimately, the Eleventh Circuit Court of Appeals (Schindler v. Schiavo, 2005) determined that Ms. Schiavo had not experienced disability discrimination, because her husband’s decision to remove her feeding tube was not based upon her disability, but upon her prior wishes.

At the same time, it is likely true that Ms. Schiavo’s HCPs were more likely than most SDMs to view her life in PVS as worse than death. It is entirely possible that Ms. Schiavo’s HCPs found it distressing to care for her, because they believed her life was not worth living. Evidence shows that HCPs often judge disabling conditions such as PVS as an existence that is worse than death. A survey sent to neurologists and medical directors in 1995, shortly before the Schiavo case made headline news, revealed that most believed patients in PVS would be better off dead (Payne et al., 1996). According to Fiester, little has changed in HCP attitudes (Ackerman, 2006; Epstein et al., 2019; Morley & Sankary, 2024), lending credibility to her position that conflicts over end-of-life care persist because that HCP values are misaligned with those of many SDMs.

It is not merely persons in vegetative states who experience this kind of disability discrimination. Many people with physical disabilities also report being told that they would be better off dead by HCPs (Carel, 2016). Fiester quotes William Peace’s experience of a physician who suggests he should refuse antibiotics and die rather than continue to live with paralysis (Peace, 2012, pp. 14–15). This is indeed a horrific if not surprising anecdote. Yet Peace’s preference to live with paralysis does not, therefore, mean that he believed there is no state of life worse than death. Implying as much, undercuts the value of the social model of disability articulated by many disability scholars. In contrast to a medical model of disability that positions disability as an individual, biological problem, the social model of disability sees the harms of disability as coming primarily through social structures and attitudes (Mukherjee et al., 2022). Peace’s description of his physicians’ biases mostly describes the social harms that can accompany paralysis (e.g., nursing home placement, unemployment, bankruptcy), many of which can be remedied by improved social policies. As some disability scholars have argued, highlighting the social indignities that disabled people experience does not suggest that there is no amount of pain or suffering a disabled person would judge as worse than death (Shakespeare, 2010). Once again, it is important not to confuse critiques over disability discrimination and quality of life misevaluations with a preference for vitalism.

## Pain Control, Vitalism, and the Law

While many disability rights organizations argued in favor of artificially feeding Terri Schiavo, many disability bioethicists have argued retrospectively that the court ultimately made the right decision to allow her surrogate decision maker to withdraw treatment (Francis & Silvers, 2007; Ouellette, 2004; Shepherd, 2006). To have overridden what the court determined to be Ms. Schiavo’s clear and convincing preference for not living life in PVS in the name of protecting her from disability discrimination would have been “a moralistic form of paternalism” according to Francis and Silvers. Moralistic paternalism “is not beneficial for people with disabilities: it imposes a view of the good on them while ignoring who they actually are in the process” (Francis & Silvers, 2007, p. 817).

Yet, there are many cases when patients with disabilities do not have clear prior directives for their care. Most state statutes require there is a “preponderance of evidence” that a patient would reject life sustaining treatments, but several states set the bar even higher, requiring SDMs to provide “clear and convincing evidence of an incompetent individual’s desire to withdraw life-sustaining treatment” including artificial hydration and nutrition (Capron, 2019; Cruzan v. Director, 1990). In cases in which a patient has never had decision-making capacity due to an intellectual disability, state legislatures will often allow SDMs and HCPs to determine which life sustaining treatments are in the patient’s “best interest.” In some rare cases, however, HCPs have been obligated to provide life sustaining treatment to incompetent patients, even when they judged treatment to be painful or even inhumane. In the case of Shelia Pouliot, which I detail below, both HCPs and SDMs agreed to withdraw life sustaining treatments, but New York state officials prevented them from doing so, based on their interpretation of New York state law. The case of Shelia Pouliot offers another cautionary tale for why it is problematic to assume a vitalistic preference on behalf of people with disabilities.

Disability bioethicists have been critical of the paternalism sometimes exercised by HCPs and the vitalism of SDMs, but conceptions of the good life can also be built into the legal system in ways that can both protect and potentially harm disabled people. In terms of Fiester’s competing BIV and LCV hierarchies, state statutes can enshrine the values of each hierarchy. For example, state statutes sometimes disallow SDMs from withdrawing or limiting pain control at the end of life. Fiester expresses this BIV value as follows: “for incapacitated patients, eliminating or minimizing physical pain is an overriding clinical obligation, even if it conflicts with other interests” (2025, p. 448). Fiester notes that surrogates sometimes place certain goods, such as interacting with a patient one final time, above maximizing pain control (2025, p. 448). Texas law, however, does not authorize physicians to withhold or withdraw pain management interventions at the end of life, even when other life sustaining treatments have been determined to be medically inappropriate (Health and Safety Code, 1999, 166.002.10). Many physicians have interpreted this statute as requiring them to reject attempts by SDMs to limit pain control at the end of life, even if other values are at stake. Adequate pain control, therefore, is interpreted by many HCPs as a legal standard that cannot be overridden by surrogates.

On the other hand, state statutes often uphold Fiester’s Life-Continuation Values by preventing HCPs from withholding or withdrawing life sustaining treatments for intellectually disabled patients. While pro-life advocates were arguing that disabled people like Terri Schiavo should always receive artificial feeding to prolong their lives, Shelia Pouliot, a profoundly disabled, terminally ill woman was artificially fed over the objections of both her medical care team and surrogate. The treatment she received has been dubbed by at least one disability legal scholar as “torture” (Ouellette, 2004, p. 1) and others have described it as a tragedy (Allen, 2006; Shepherd, 2006).

In 1999, Pouliot was admitted to the State University of New York Upstate Medical Center at Syracuse for bleeding in her intestinal tract and she could no longer tolerate feeding through her gastrostomy tube. Pouliot could not tolerate the typical 900 cal a day, which caused projectile vomiting, and instead was given 300 cal of sugar water with no protein, which technically prolonged her life but according to her treating physician the treatment caused her body to catabolize tissue, her muscles to rot, her heart to deteriorate, and her skin to break down (Faber-Langendoen, 2000). Pouliot’s physicians agreed she was in considerable pain from her terminal illness. In the final months of her life, she was seen to be crying, moaning, and grimacing from the half measures of artificial nutrition and hydration she was given (Shepherd, 2006, p. 271).

Both Ms. Pouliot’s HCPs and SDM agreed that it was in her best interest to remove her feeding tube, but the New York Attorney General’s office intervened and advised hospital officials that New York law required the continuation of Ms. Pouliot’s artificial nutrition and hydration because no third party could authorize the withdrawal. Because Ms. Pouliot had never been competent, she could have never consented to the withdrawal of life sustaining treatment. The hospital, therefore, was legally required to administer artificial hydration and nutrition under New York law (*Blouin ex Rel. Estate of Pouliot v. Spitzer*, 2004). According to her treating physician, the result was that Ms. Pouliot died a “slow lingering death from protein malnutrition” (Faber-Langendoen, 2000). Disability legal scholar Alicia Ouellette concludes that “Sheila Pouliot was a victim of vitalism run amuck” and that state laws should “allow surrogates to make medical decisions based in part on the quality of life of the patient” (2004, pp. 21–22).

After much publicity surrounding Ms. Pouliot’s case, New York enacted new legislation that allows guardians of intellectually disabled people to withhold life-sustaining treatment if it has “no reasonable hope of maintaining life” or “poses an extraordinary burden,” (N.Y. Surr. CT. Proc. Act *§* 1750-b). Ouellette point out, however, that there are several states throughout the country where cases like that of Sheila Pouliot could be repeated because they require the administration of artificial hydration and nutrition at the end of life if a patient does not have an advance directive or clear prior statement indicating they would reject it, which is impossible for patients who have always been incompetent (Ouellette, 2004). Twenty years after Ouellette issued this warning, little has changed in most of these state statutes. States that build vitalism into their legal statues risk doing harm to intellectually disabled people who cannot make their own medical decisions.

Had Ms. Pouliot lived in a state with no artificial feeding requirement, but the same treatment plan was demanded by her surrogates, the result would have been equally insupportable. Although many disability rights advocates seem to uphold LCV values when they argue that QoL judgments should not be made to determine who lives or dies, Ouellette compellingly argues that in cases similar to Shelia Pouliot, QoL should be taken into consideration. HCPs should and often do find it ethically intolerable to cause a patient considerable pain at the end of life through forced medical treatment. Quality of life considerations in the case of Ms. Pouliot are a far cry from the judgment that a person with a brain injury or any other disability is “better off dead.” HCPs and bioethicists should be aware of their own potential implicit biases against disability, but this does not mean that disabled patients must be maximally treated regardless of their prior wishes or the burdens that treatment can impose. Forcing treatment on intellectually disabled people simply because they do not have the autonomy to reject it amounts to disability discrimination.

## Conclusion

Disability rights organizations have not attended to the risks of vitalism for people with disabilities as thoroughly as QoL misevaluations. At present, QoL determinations are likely a more pressing issue for many disabled people, who, like William Peace, have experienced this kind of discrimination from HCPs first-hand. Yet, disability discrimination can happen through limiting as well as administering medical treatments at the end of life. Many disability advocates who were worried that Terri Schiavo was being denied life sustaining treatment because of her disability supported right to life model legislation to ensure that no disabled person could be denied artificial hydration and nutrition at the end of life without a written or oral advance directive (NCIL, 2005; NRLC, 2006). Yet, patients like Shelia Pouliot could never have such directives and will bear the cost of such legislation.

Parsing quality of life misevaluations from vitalism is a difficult but important task for HCPs and bioethicists. Surrogates who demand aggressive treatment for a disabled patient may be protecting their interests from the biases of HCPs, but they may also be pursuing their own values and interests. From the HCP’s perspective, a surrogate who advocates for continuing life sustaining treatment in the face of a patient’s clear debility or poor prognosis may appear to hold vitalist values. What an HCP judges to be in the “best interest” of the patient, may not align well with a surrogate’s judgment of what kind of life the patient would find worth living. A preference for continuing disabled life, however, should not be confused with a belief that any state of life is preferable to death. Most disabled people can imagine a future life that is worse than death, and treatments they would rather not endure for the sake of prolonging life. Such considerations become even more complex for patients who have never been able to articulate their preferences for medical treatment. Health care practitioners are thus left with the difficult task of weighing surrogate requests life sustaining treatments for disabled patients with the potential or foreseen harms of those treatments. In cases such as those of Ms. Pouliot, HCPs should not be pressured by SDM or governments to provide end-of-life treatments that cause considerable pain for the prospect of a few more days or weeks of life.

Of course, surrogates are often in the best position to understand the lives, experiences, and needs of disabled patients, and for this reason, HCPs should often defer to their judgment regarding the patient’s QoL as well as their end-of-life care. However, there are times when such deference ought to be critically assessed or even rejected. Attempting to protect disabled patients, surrogates and state laws can exercise unacceptable paternalism in the name of Life- Continuation Values that hurt rather than help disabled patients. Of course, understanding when to defer to surrogates and when to override them will require considerable education around the lived experiences of people with disabilities or what some have dubbed “disability cultural competency,” which has not yet found a foothold in bioethics or medical education (Garland-Thomson & Iezzoni, 2021; Mukherjee et al., 2022). If HCPs wish to uphold their values and serve their disabled patients, such education is critical.

## Notes

It is worth noting in this case that Mr. Hickson had a court appointed guardian and so Mrs. Hickson was not his legal decision-maker. Public reporting on the case has not made it clear why a Texas probate court appointed an elder care agency to make Mr. Hickson’s medical decisions when he had a competent next-of-kin caretaker willing and able to make decisions on his behalf. Mrs. Hickson claimed it was because she disagreed with another hospital’s discharge plan for her husband (Shapiro, 2020). According to a press release from Mrs. Hickson’s lawyers who sued the elder care agency that provided Mr. Hickson’s guardian, the guardian assigned to Mr. Hickson followed the recommendation given by his physician to withdraw life-sustaining treatment without question and instructed the hospital’s social worker to withhold medical information from Mrs. Hickson (Robbins Di Monte, 2021).
